# Altered amyloid precursor protein processing regulates glucose uptake and oxidation in cultured rodent myotubes

**DOI:** 10.1007/s00125-014-3269-x

**Published:** 2014-05-22

**Authors:** D. Lee Hamilton, John A. Findlay, Gemma Montagut, Paul J. Meakin, Dawn Bestow, Susan M. Jalicy, Michael L. J. Ashford

**Affiliations:** Division of Cardiovascular and Diabetes Medicine, Medical Research Institute, Ninewells Hospital & Medical School, University of Dundee, Dundee, DD1 9SY Scotland UK

**Keywords:** Amyloid, BACE1, Glucose uptake, Glut4, Insulin, PI3K, Skeletal muscle, Type 2 diabetes

## Abstract

**Aims/hypothesis:**

Impaired glucose uptake in skeletal muscle is an important contributor to glucose intolerance in type 2 diabetes. The aspartate protease, beta-site APP-cleaving enzyme 1 (BACE1), a critical regulator of amyloid precursor protein (APP) processing, modulates in vivo glucose disposal and insulin sensitivity in mice. Insulin-independent pathways to stimulate glucose uptake and GLUT4 translocation may offer alternative therapeutic avenues for the treatment of diabetes. We therefore addressed whether BACE1 activity, via APP processing, in skeletal muscle modifies glucose uptake and oxidation independently of insulin.

**Methods:**

Skeletal muscle cell lines were used to investigate the effects of BACE1 and α-secretase inhibition and BACE1 and APP overexpression on glucose uptake, GLUT4 cell surface translocation, glucose oxidation and cellular respiration.

**Results:**

In the absence of insulin, reduction of BACE1 activity increased glucose uptake and oxidation, GLUT4myc cell surface translocation, and basal rate of oxygen consumption. In contrast, overexpressing BACE1 in C_2_C_12_ myotubes decreased glucose uptake, glucose oxidation and oxygen consumption rate. APP overexpression increased and α-secretase inhibition decreased glucose uptake in C_2_C_12_ myotubes. The increase in glucose uptake elicited by BACE1 inhibition is dependent on phosphoinositide 3-kinase (PI3K) and mimicked by soluble APPα (sAPPα).

**Conclusions/interpretation:**

Inhibition of muscle BACE1 activity increases insulin-independent, PI3K-dependent glucose uptake and cell surface translocation of GLUT4. As APP overexpression raises basal glucose uptake, and direct application of sAPPα increases PI3K–protein kinase B signalling and glucose uptake in myotubes, we suggest that α-secretase-dependent shedding of sAPPα regulates insulin-independent glucose uptake in skeletal muscle.

## Introduction

A fundamental characteristic of type 2 diabetes is the occurrence of insulin resistance in liver, adipose tissue and skeletal muscle. However, the first detectable defect in individuals predisposed to type 2 diabetes is altered responsiveness of skeletal muscle to insulin [1, 2], the principal site for insulin-stimulated glucose disposal [3]. Glucose transport is the rate-limiting step for glucose metabolism in healthy and diabetic individuals [4, 5], with decreased insulin-stimulated glucose uptake into skeletal muscle being a major contributory factor to insulin resistance in patients with type 2 diabetes [6–8]. Insulin promotes glucose transport in skeletal muscle by stimulating the translocation of the insulin-responsive glucose transporter, GLUT4, from intracellular storage vesicles to the plasma membrane [9]. In insulin-resistant humans, normal total expression of GLUT4 is reported [10], but with decreased cell surface levels [11, 12] due to reduced translocation of GLUT4-containing vesicles [13]. Furthermore, muscle-specific deletion of GLUT4 causes glucose intolerance and diabetes in mice [14] with reduced insulin-stimulated glucose uptake [15]. Conversely, muscle-specific overexpression of GLUT4 improves insulin action and decreases plasma glucose levels in diabetic mice [16].

Some studies have linked type 2 diabetes to increased risk of Alzheimer’s disease (AD), with AD patients being more prone to impaired glucose metabolism, hyperinsulinaemia and insulin resistance [17, 18]. A key feature of AD development is enhanced proteolytic cleavage of the amyloid precursor protein (APP) by the aspartyl protease, beta-site APP-cleaving enzyme 1 (BACE1), which, with γ-secretase, raises levels of β-amyloid peptides leading to amyloid aggregation and plaque formation [19]. However, cleavage of APP by α-secretases (e.g. a disintegrin and metalloprotease [ADAM] family member) predominates under basal conditions. BACE1 and α-secretase are sheddases, cleaving the extracellular portions of APP and releasing their ectodomains (soluble [s]APPβ and sAPPα, respectively) from the cell surface. These soluble ectodomains may have physiological functions, with sAPPα being neuroprotective and able to modulate cognitive performance and neuronal plasticity [20].

Reduction or loss of BACE1 by genetic manipulation in mice improves glucose disposal and insulin sensitivity on regular and high-fat diets, and pharmacological inhibition of BACE1 in mouse C_2_C_12_ myotubes increases insulin sensitivity [21]. In addition, BACE1 inhibition or overexpression in C_2_C_12_ myotubes increases or decreases, respectively, insulin-stimulated glucose uptake. This suggests that skeletal muscle BACE1 activity is important in the regulation of insulin-driven glucose disposal, but the mechanism by which this occurs is unknown. Skeletal muscle glucose uptake has insulin-dependent and insulin-independent components [22, 23]. Furthermore, in skeletal muscle from patients with type 2 diabetes, the insulin resistance associated with diminished glucose uptake is probably due to post-receptor defect(s), with impaired GLUT4 translocation to the plasma membrane [11, 23, 24]. As current therapies aimed at alleviating insulin resistance and improving glucose uptake are insufficient and often incompletely effective, there is an urgent need for information on novel pathways that modulate GLUT4 translocation and glucose uptake which are amenable to pharmacological intervention.

Consequently, we examined whether BACE1 activity and APP processing in skeletal muscle modulates glucose uptake primarily through altered insulin sensitivity or is capable of engaging an insulin-independent pathway.

## Methods

### Cell culture

L6-GLUT4myc rat myoblasts (kindly provided by Amira Klip, Toronto, Canada) were maintained in α-minimum essential media (MEM) with 10% FBS. C_2_C_12_ and L6-GLUT4myc myoblasts were differentiated to myotubes as described previously [21]. C_2_C_12_ cells were transfected with 12 μg DNA of empty vector (EV; pcDNA3.1) or pcDNA3.1 containing full-length BACE1, BACE1 active site mutant (mBACE1 [25]) or APP using Lipofectamine 2000 (Invitrogen Life Technologies, Paisley, UK). Cells were selected with 1 mg/ml G418 (Sigma–Aldrich, Gillingham, UK) and differentiated. A minimum of two independently generated stable cell lines with concurrently produced EV controls was used. After 4 days of differentiation, cells were treated with TAPI-1 (Invitrogen), Merck-3 (β-secretase inhibitor IV [M-3]), BACE1 inhibitor II, batimastat, palmitate or d-erythro-sphingosine, *N*-acetyl-C2-ceramide (all Calbiochem, Nottingham, UK), or appropriate vehicle, overnight (∼20 h).

### Cloning

Full-length myc-his human BACE1 in pcDNA3.1 was obtained from GlaxoSmithKline (Harlow, UK). mBACE1 (a gift from Professor Wolfe [Brigham and Women’s Hospital, Boston, MA, USA]) was subcloned into pcDNA3.1. Full-length human APP (Genbank AAH65529.1) was amplified with primers APP Fwd: 5'-AAAGCTAGCATGCTGCCCGGTTTG-3'; APP Rev: 5'-TTTAAGCTTCTAGTTCTGCATCTGCTCAAA-3' and cloned into pcDNA3.1.

### Immunoblotting and gene expression

Protein isolation and immunoblotting procedures were as described previously [22]. For quantification of sAPP fragments, differentiated cells were treated in 10 ml Optimem (Gibco Life Technologies, Paisley, UK), and media were concentrated (30 kDa Amicon Ultra 15 ml filter) by centrifugation (4,000 *g*) and subjected to SDS-PAGE with amounts presented relative to total protein. Primary antibodies used were: anti-sAPPβ (Covance, Alnwick, UK; 1:1,000), anti-sAPPα (IBL International, Hamburg, Germany; 1:50), anti-APP (Ab54, GlaxoSmithKline; 1:4,000), anti-phospho-PKB (protein kinase B; Ser^473^), anti-PKB and anti-HKII (hexokinase II) (Cell Signaling, Hitchin, UK; 1:1,000), anti-GLUT1 (Millipore, Nottingham, UK; 1:1,000) and anti-GLUT4 (Abcam, Cambridge, UK; 1:1,000). *Glut1* (also known as *Slc2a1*), *Glut4* (also known as *Slc2a4*) and *HkII* (also known as *Hk2*) mRNA was determined by TaqMan RT-PCR (Applied Biosystems, Paisley, UK; Prism Model 7700) using commercial primers and probe sets.

### Cell surface GLUT4myc detection

For details, see Wang et al [26]. Briefly, L6-GLUT4myc myoblasts or myotubes were serum-starved (4 h), treated with M-3 (250 nmol/l), stimulated with insulin (20 or 100 nmol/l) or vehicle at 37°C for 30 min, and fixed in 3% paraformaldehyde. Anti-c-myc (A14; Santa Cruz, Heidelberg, Germany; 1:100) primary antibody was applied (1 h), and peroxidase-conjugated rabbit anti-mouse secondary antibody (1:1,000) in 3% goat serum was added, followed by 1 ml *o*-phenylenediamine dihydrochloride (OPD reagent; Sigma), and incubated (in the dark) for 30 min. The reaction was stopped with 250 μl 3 mol/l HCl, and absorbance of the supernatant fraction was measured.

### Glucose uptake

C_2_C_12_ myotubes, stably expressing BACE1, APP or mBACE1, or EV controls were exposed to M-3 (250 nmol/l), BACE1 inhibitor II (0.7 μmol/l), palmitate (750 μmol/l), ceramide (50 μmol/l) or batimastat (5 μmol/l). Cells were serum-starved (2 h) and exposed to insulin (100 nmol/l), sAPPα (0.3, 3 or 10 nmol/l), sAPPβ (0.3, 3 or 10 nmol/l) or vehicle control. For phosphoinositide 3-kinase (PI3K)-dependence, myotubes were pretreated overnight with M-3 or vehicle, then with wortmannin (100 nmol/l) for 1 h before insulin. Myotubes were incubated (12 min) with 10 μmol/l 2-deoxy-d-[^3^H]glucose (2DG; 24.4 kBq/ml; PerkinElmer, Cambridge, UK) at 20°C. Non-specific uptake was determined using 10 μmol/l cytochalasin B (Sigma–Aldrich). After lysis, cell-associated radioactivity was measured (Beckman, High Wycombe, UK; LS 6000IC scintillation counter), and protein was quantified using the Bradford reagent.

### Oxidation assays

C_2_C_12_ myotubes pre-exposed to M-3 (250 nmol/l), BACE1 inhibitor II (0.7 μmol/l) or batimastat (5 μmol/l) were incubated with 5 mmol/l glucose and 74 kBq/ml d-[U-^14^C]glucose (PerkinElmer) for 2 h at 37°C (glucose oxidation assay) or 5 mmol/l glucose and 750 μmol/l palmitate (conjugated to BSA)/[^14^C]palmitate (74 kBq/ml) for 3 h (palmitate oxidation assay). Medium was transferred to 15 ml centrifuge tubes, ^14^CO_2_ released using 60% (v/v) perchloric acid was trapped by a Whatman (GF/B) filter paper disc presoaked with 1 mol/l KOH, and radioactivity was quantified by liquid-scintillation counting.

### Immunostaining and imaging

C_2_C_12_ myotubes were fixed in 4% paraformaldehyde, permeabilised in PBST (PBS + 0.1% Triton X-100), blocked in 10% donkey serum (Sigma–Aldrich) and then incubated with primary antibodies: anti-APP (Ab54, 1:100) or anti-BACE1 (Sigma–Aldrich, 1:250). Secondary antibodies were Cy3 (Jackson ImmunoResearch, Newmarket, UK; 1:250) and Alexa Fluor 488 (Invitrogen, 1:250). Images were acquired with a confocal laser-scanning microscope (Leica, Milton Keynes, UK; TCS SP5 II).

### Cellular respiration

Myoblasts were seeded in XF 24-well culture microplates (Seahorse Bioscience, Copenhagen, Denmark), differentiated for 4 days and changed to Opti-MEM-reduced serum medium incubated at 37°C (24 h). Cells were washed in Krebs–Henseleit buffer (in mmol/l: 111 NaCl, 4.7 KCl, 2 MgSO_4_.7H_2_O and 1.2 Na_2_HPO_4_) containing 5 mmol/l glucose and 0.5 mmol/l l-carnitine, and baseline recordings were taken to ensure a steady respiratory rate before injection of palmitate (750 μmol/l).

### Statistical analysis

Comparisons between groups were performed using an unpaired two-tailed Student’s *t* test, one-sample Student’s *t* test or ANOVA with repeated measures and Tukey’s multiple comparison test, as appropriate, using GraphPad (Prism 5) software (GraphPad Software, La Jolla, CA, USA). *p* values ≤ 0.05 were considered significant.

## Results

### Glucose uptake and GLUT4 translocation in myotubes are modulated by BACE1 activity in an insulin-independent manner

**Fig. 1:**
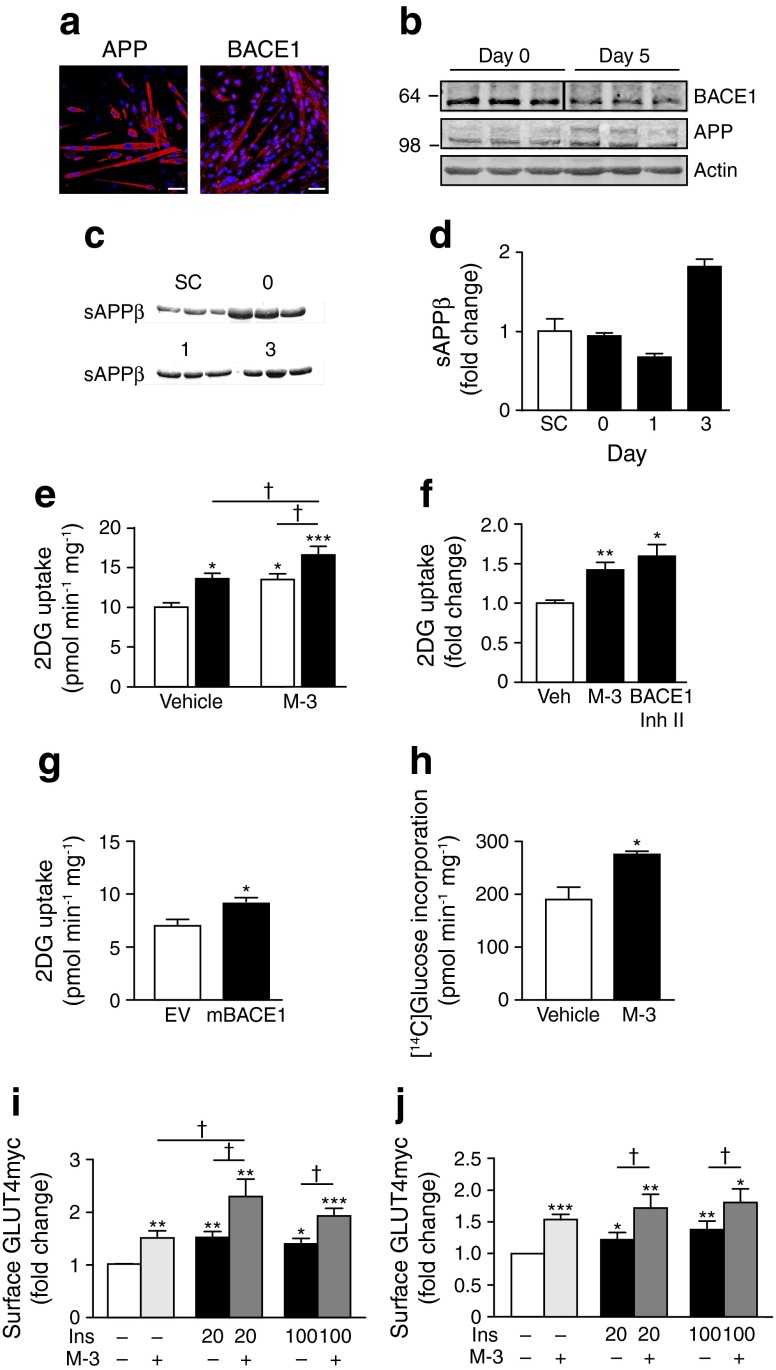
BACE1 inhibition increases glucose uptake and GLUT4 translocation. (**a**) Immunohistochemistry for APP and BACE1 in C_2_C_12_ myotubes. Scale bar, 50 μm. (**b**) Representative immunoblots of BACE1 and APP in C_2_C_12_ myoblasts in differentiation medium on days 0 (myoblasts) and 5 (myotubes). (**c**) sAPPβ in the medium of C_2_C_12_ myoblasts (day −1 [SC; sub-confluent] and 0) and myotubes (days 1 and 3). (**d**) Quantification of sAPPβ (relative to total protein) before (−1) and during differentiation. (**e**) Basal and insulin-stimulated 2-deoxyglucose (2DG) uptake in control and M-3-treated myotubes (*n* = 11); white bars, vehicle; black bars, insulin. (**f**) 2DG uptake in myotubes treated with vehicle (Veh), M-3 or BACE1 inhibitor (Inh) II (*n* = 6–11). (**g**) 2DG uptake in myotubes transfected with EV or mBACE1 (*n* = 4). (**h**) Total [^14^C]glucose incorporation in myotubes after vehicle and M-3 (*n* = 4). (**i**, **j**) Gain in cell surface GLUT4myc ± insulin (Ins; 20 and 100 nmol/l) ± M-3 in myotubes (**i**) and myoblasts (**j**) (*n* = 4). *p < 0.05, **p < 0.01, ***p < 0.001 vs vehicle alone; †*p* < 0.05

We detected BACE1 and APP protein in wild-type C_2_C_12_ myoblasts and myotubes (Fig. 1a, b) and demonstrated that BACE1 was proteolytically active by the presence of sAPPβ in the incubation medium (Fig. 1c, d). Inhibition of BACE1 activity by application of M-3 [27] to myotubes before challenge with 100 nmol/l insulin increased insulin-stimulated glucose uptake compared with insulin alone (Fig. 1e). However, M-3, in the absence of insulin also increased glucose uptake (Fig. 1e, f). To confirm that this effect was mediated by BACE1 inhibition, we treated wild-type myotubes with a structurally dissimilar BACE1 inhibitor (BACE1 inhibitor II), which increased glucose uptake (Fig. 1f). Overexpression of mBACE1, which is devoid of protease activity [25], also increased glucose uptake (Fig. 1g). We also detected increased [^14^C]glucose incorporation in M-3-treated C_2_C_12_ myotubes (Fig. 1h).

**Fig. 2:**
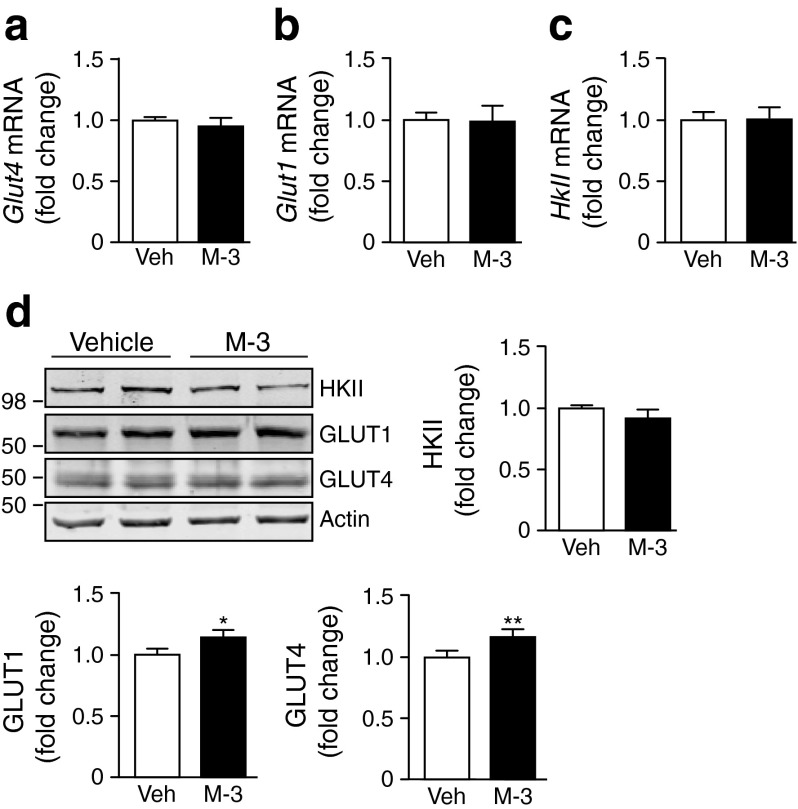
BACE1 inhibition modifies expression of glucose transporters. Quantitative PCR analysis of mRNA for (**a**) *Glut4*, (**b**) *Glut1* and (**c**) *HkII* in control and M-3-treated myotubes. (**d**) Representative immunoblots of HKII, GLUT1 and GLUT4 in control and M-3-treated myotubes, with quantification of the immunoblot data shown (*n* = 8). Veh, vehicle. *p = 0.05, **p < 0.01 vs vehicle alone

To examine the effect of BACE1 inhibition on plasma membrane GLUT4 levels, we used rat differentiated L6 myotubes overexpressing GLUT4 with an exofacial myc-epitope tag (GLUT4myc) [26]. Insulin (20 nmol/l) stimulation of myotubes induced a gain in cell surface GLUT4myc (Fig. 1i), as expected [27]. However, M-3 in the absence of insulin also caused a gain in cell surface GLUT4myc, with insulin (20 nmol/l) and M-3 producing an additive outcome (Fig. 1i). Exposure of myotubes to a supramaximal insulin concentration (100 nmol/l) in the absence and presence of M-3 resulted in a gain of cell surface GLUT4myc, individually and additively indistinguishable from the 20 nmol/l insulin experiments (Fig. 1i). M-3 treatment of GLUT4myc myoblasts also resulted in enhancement of an insulin-dependent and insulin-independent gain in cell surface GLUT4myc (Fig. 1j). These results strongly indicate that BACE1 inhibition increases basal glucose uptake through an insulin-independent pathway. M-3 had no effect on *Glut4*, *Glut1* or *HkII* mRNA expression (Fig. 2a–c) or HKII protein levels, but modestly increased GLUT1 and GLUT4 levels in C_2_C_12_ myotubes (Fig. 2d).

### Palmitate and ceramide increase BACE1 and inhibit glucose uptake

**Fig. 3:**
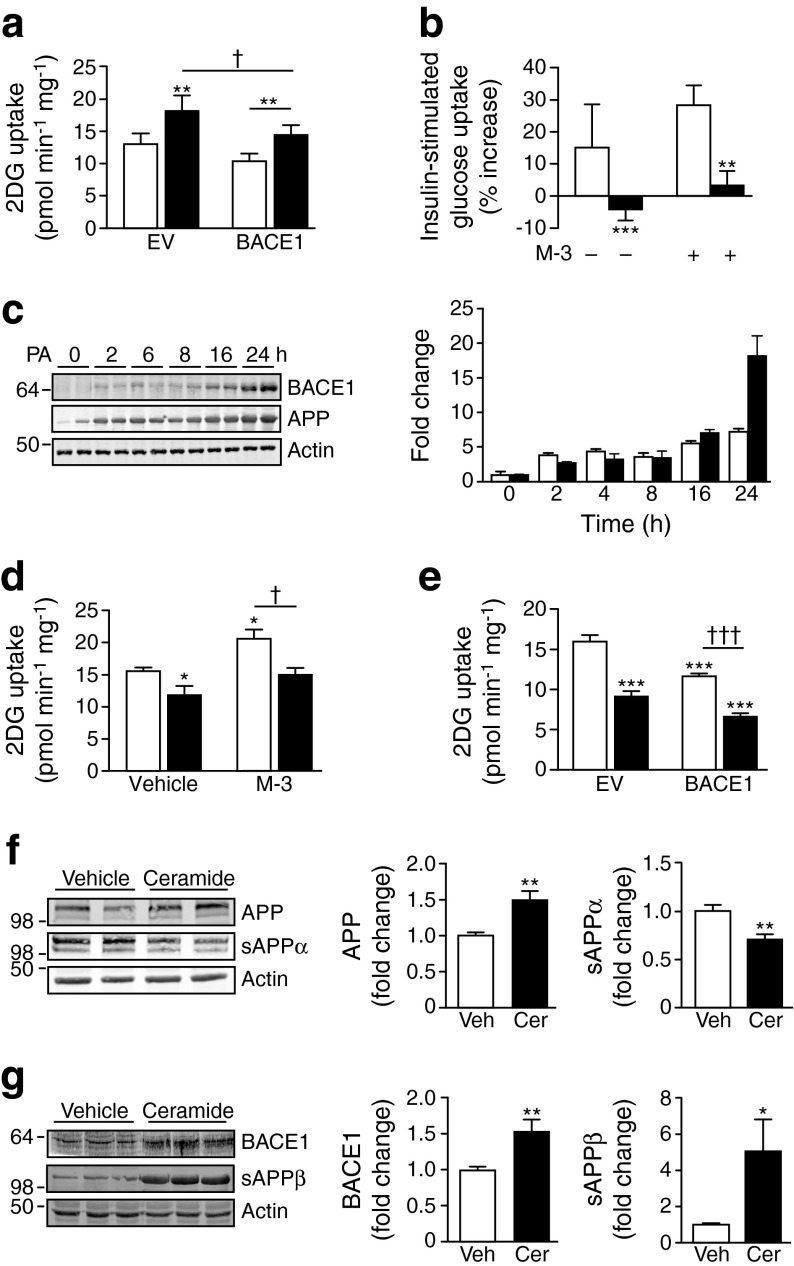
Raised BACE1 and exposure to palmitate and ceramide reduces basal glucose uptake. (**a**) 2DG uptake in myotubes transfected with EV or BACE1 ± insulin (100 nmol/l; *n* = 12); white bars, vehicle; black bars, insulin. (**b**) Palmitate inhibits insulin-stimulated glucose uptake in the absence and presence of M-3; white bars, vehicle; black bars, palmitate. (**c**) Immunoblot and bar graph (white bars, APP; black bars, BACE1) showing the effect of palmitate on APP and BACE1 levels. 2DG uptake in myotubes (**d**) treated with vehicle or M-3 ± ceramide (50 μmol/l; *n* = 4–7) or (**e**) transfected with EV or BACE1 ± ceramide (50 μmol/l; *n* = 4); white bars, vehicle; black bars, ceramide. Representative immunoblots of (**f**) APP (*n* = 8) and sAPPα (*n* = 9–12) and (**g**) BACE1 (*n* = 8) and sAPPβ (*n* = 9–12) from myotubes treated with vehicle (Veh) or ceramide (Cer; 50 μmol/l), with mean data represented graphically. *p < 0.05, **p < 0.01, ***p < 0.001 vs vehicle or EV alone; ^†^ *p* < 0.05, ^†††^ *p* < 0.001

Overexpression of BACE1 decreased insulin-stimulated glucose uptake (Fig. 3a) and depressed basal glucose uptake in C_2_C_12_ myotubes (Figs 3a, e and 5c). To address whether manipulating BACE1 could affect glucose uptake in the context of metabolic distress, we challenged C_2_C_12_ myotubes with the saturated fatty acid, palmitate. Palmitate (750 μmol/l) inhibited insulin-stimulated glucose uptake in the absence and presence of M-3 (Fig. 3b), although this was associated with substantially increased BACE1 and APP protein levels (Fig. 3c). As the palmitate metabolite, ceramide, mimics the effects of saturated fatty acid oversupply on insulin sensitivity [28] and ceramide content is raised in skeletal muscle of insulin-resistant rodents and humans [29, 30], we decided to use this molecule to further examine M-3-sensitive glucose uptake. Incubation of myotubes with ceramide (50 μmol/l) decreased basal and M-3-stimulated glucose uptake (Fig. 3d) and further suppressed glucose uptake inhibited by BACE1 overexpression (Fig. 3e). However, ceramide also increased myotube APP and BACE1 levels with decreased sAPPα and increased sAPPβ levels (Fig. 3f, g). These findings indicate that ceramide also inhibits the BACE1-dependent, insulin-independent pathway of glucose uptake.

### BACE1 activity alters substrate oxidation of C_2_C_12_ myotubes

**Fig. 4:**
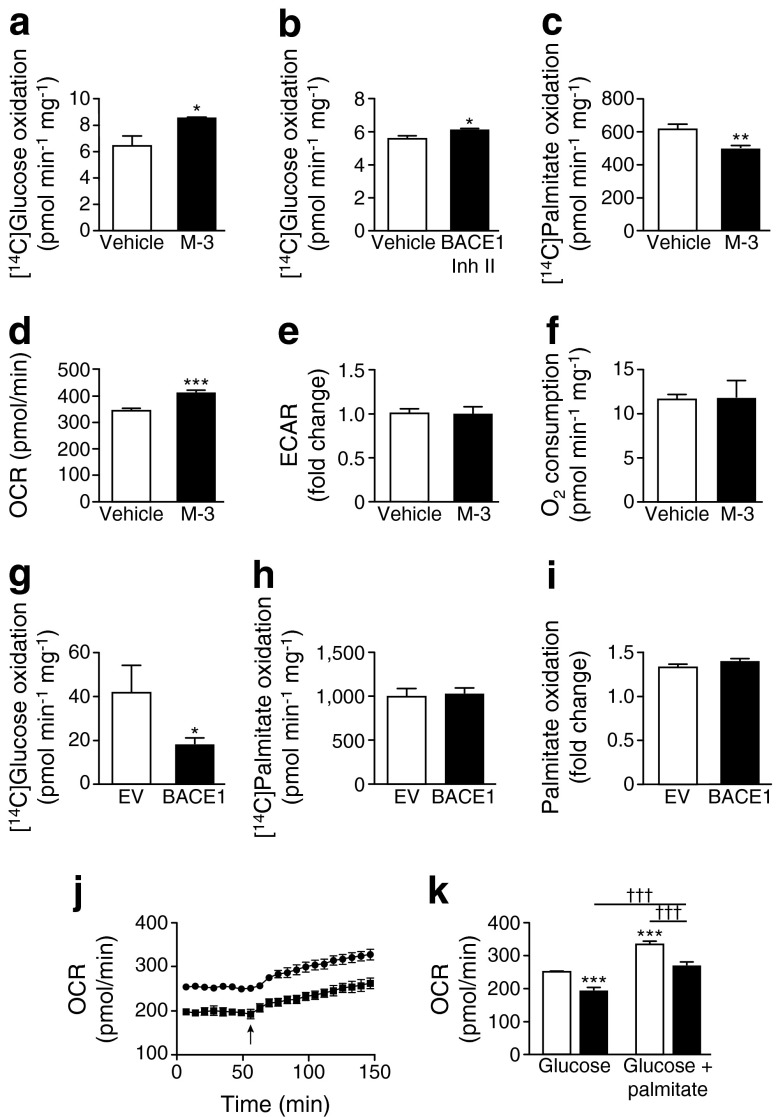
Increased BACE1 reduces glucose oxidation. (**a**, **b**) [^14^C]Glucose oxidation of cells in vehicle and M-3 (**a**) and BACE1 inhibitor (Inh) II (**b**) (*n* = 4). (**c**) [^14^C]Palmitate oxidation of cells in vehicle and M-3 (*n* = 4). OCR (**d**) and extracellular acidification rate (ECAR) (**e**) of myotubes ± M-3 (*n* = 9). (**f**) Oxygen consumption for glucose + palmitate ± M-3 (*n* = 3). [^14^C]Glucose (**g**) and [^14^C]palmitate (**h**) oxidation in myotubes transfected with EV or BACE1 (*n* = 12). (**i**) Relative increase in palmitate oxidation in myotubes transfected with EV or BACE1 (*n* = 4). (**j**) OCR of EV- and BACE1-transfected myotubes in the presence of glucose and glucose + palmitate (arrow; *n* = 4); circles, EV; squares, BACE1. (**k**) OCR of myotubes transfected with EV and BACE1 in the presence of glucose and glucose + palmitate (*n* = 4); white bars, EV; black bars, BACE1. *p < 0.05, **p < 0.01, ***p < 0.001 vs vehicle or EV alone; ^†††^ *p* < 0.001

The increased glucose uptake in skeletal muscle cells by BACE1 inhibition was accompanied by increased glucose oxidation (Fig. 4a, b), whereas BACE1 inhibition reduced fatty acid (palmitate) oxidation (Fig. 4c). Real-time analysis of the oxygen consumption rate (OCR) by C_2_C_12_ myotubes confirmed that M-3 raised basal glucose oxidation, with no change in glycolysis (Fig. 4d, e). Interestingly, M-3 had no effect on myotube oxygen consumption in the presence of glucose and palmitate (Fig. 4f), indicating that decreased BACE1 activity drives substrate switching to oxidise a greater proportion of glucose over fatty acids to maintain ATP production. Overexpression of BACE1 reduced glucose oxidation (Fig. 4g), but did not affect palmitate oxidation (Fig. 4h). These effects of increased BACE1 were confirmed by real-time OCR analysis demonstrating reduced glucose oxidation with unaltered myotube OCR in response to palmitate (Fig. 4i, j) giving an overall reduced myotube oxygen consumption for combined substrate (Fig. 4k).

### APP cleavage may mediate changes in glucose uptake by BACE1 activity

**Fig. 5:**
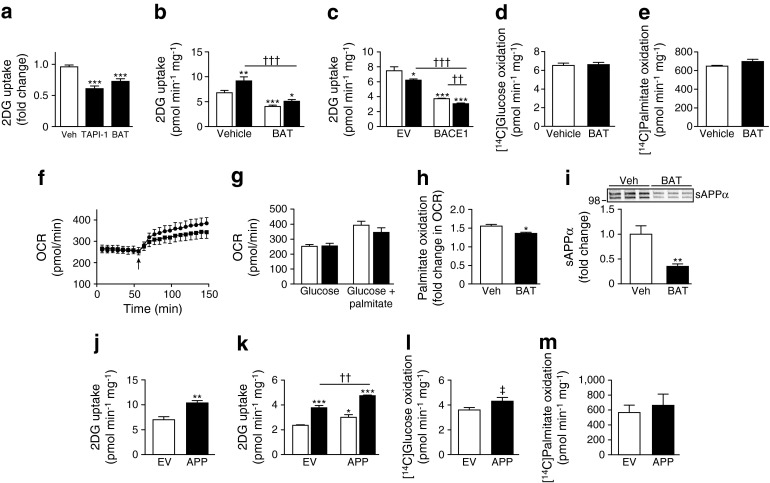
APP cleavage underlies altered basal glucose uptake. (**a**) TAPI-1 and batimastat (BAT) both reduce 2DG uptake in myotubes (*n* = 4). (**b**) Basal and insulin-stimulated 2DG uptake in vehicle and batimastat-treated myotubes (*n* = 6); white bars, vehicle; black bars, insulin. (**c**) Effect of batimastat on 2DG uptake in myotubes transfected with EV and BACE1 (*n* = 3); white bars, vehicle; black bars, batimastat. Batimastat has no effect on [^14^C]glucose (**d**; *n* = 4) or [^14^C]palmitate (**e**; *n* = 3) oxidation in myotubes. (**f**) OCR of myotubes treated with vehicle or batimastat in the presence of glucose and glucose + palmitate (arrow; *n* = 4; circles, vehicle; squares, batimastat), with mean results presented graphically in (**g**); white bars, vehicle; black bars, batimastat. (**h**) Relative increase in palmitate oxidation in myotubes treated with batimastat (*n* = 4). (**i**) Representative immunoblots of sAPPα in medium from vehicle and batimastat-treated myotubes. Histogram shows normalised data from immunoblots (*n* = 10). (**j**) Basal 2DG uptake in EV- and APP-transfected myotubes (*n* = 4). (**k**) Basal and insulin-stimulated 2DG uptake in EV- and APP-transfected myotubes (*n* = 4); white bars, vehicle; black bars, insulin. APP overexpression has no major effect on [^14^C]glucose (**l**; *n* = 4) or [^14^C]palmitate (**m**; *n* = 3) oxidation in myotubes. Veh, vehicle. *p < 0.05, **p < 0.01, ***p < 0.001 vs vehicle or EV alone; ^††^ *p* < 0.01, ^†††^ *p* < 0.001; ^‡^ *p* = 0.06

Exposure of myotubes to the α-secretase inhibitors, TAPI-1 (20 μmol/l) and batimastat (5 μmol/l [31]), depressed glucose uptake (Fig. 5a, b). Batimastat also reduced insulin-stimulated glucose uptake (Fig. 5b) and potentiated the depression of insulin-dependent and independent glucose uptake by overexpression of BACE1 (Fig. 5c). Batimastat treatment of C_2_C_12_ myotubes did not alter glucose (Fig. 5d) or palmitate (Fig. 5e) oxidation or OCR in glucose alone or glucose and palmitate (Fig. 5f, g). However, batimastat-treated myotubes showed reduced ability to switch substrate from glucose to palmitate in response to increased substrate delivery (Fig. 5h), indicating diminished metabolic flexibility. These data suggest that myotube α-secretase substrate cleavage resulted in the maintenance or enhancement of basal (insulin-independent) glucose uptake but did not affect fuel oxidation. Indeed, APP is actively cleaved in C_2_C_12_ myotubes to shed sAPPα into the medium, an effect inhibited by batimastat (Fig. 5i). A similar reduction was seen with TAPI-1 (data not shown). Overexpression of APP in C_2_C_12_ myotubes increased basal and insulin-stimulated (Fig. 5j, k) glucose uptake, but had little or no effect on glucose or palmitate oxidation (Fig. 5l, m).

### M-3-dependent glucose uptake is PI3K-dependent and mimicked by sAPPα

**Fig. 6:**
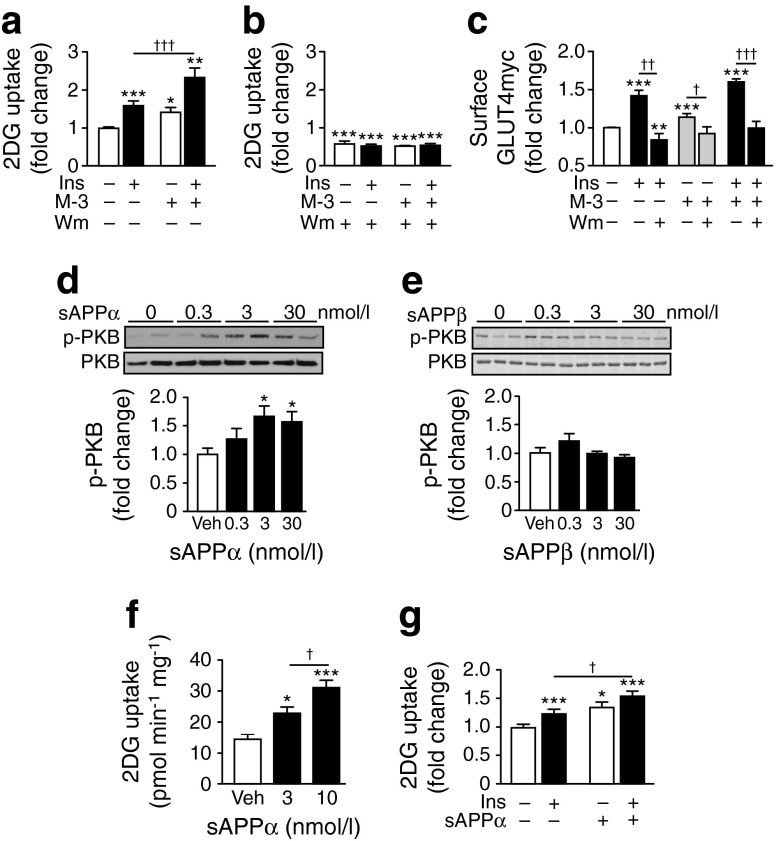
M-3-stimulated glucose uptake is PI3K-dependent and mimicked by sAPPα. Basal and insulin-stimulated 2DG uptake in vehicle and M-3-treated myotubes in the absence (**a**) and presence (**b**) of wortmannin (Wm; *n* = 6–12). (**c**) Gain in cell surface GLUT4myc in control and M-3- and insulin-treated myotubes ± wortmannin (*n* = 4–11). Representative immunoblots of sAPPα- (**d**) and sAPPβ- (**e**) stimulated PKB phosphorylation at Ser^473^ and total PKB in myotubes. Histograms show normalised means ± SEM of immunoblots (*n* = 5–6). (**f**) 2DG uptake in myotubes treated with vehicle or sAPPα (*n* = 8). (**g**) Relative increase in 2DG uptake from vehicle, insulin and sAPPα (3 nmol/l) stimulated myotubes (*n* = 14–16). Veh, vehicle. *p < 0.05, **p < 0.01, ***p < 0.001 vs vehicle; ^†^ *p* < 0.05, ^††^ *p* < 0.01, ^†††^ *p* < 0.001

Insulin-stimulated GLUT4 translocation and glucose uptake in skeletal muscle require activity of the PI3K–PKB pathway [32]. Wortmannin (100 nmol/l) blocked the increase in glucose uptake elicited by insulin (100 nmol/l), as expected, and prevented M-3 from increasing glucose uptake in C_2_C_12_ myotubes (Fig. 6a, b). Consistent with the involvement of PI3K signalling in APP/BACE1-modulated basal glucose uptake, the gain in cell surface GLUT4myc caused by M-3, in the absence and presence of insulin, was inhibited by wortmannin (Fig. 6c). To further explore the role of α- and β-secretase APP cleavage, we examined the effects of the respective cleavage products, sAPPα and sAPPβ on activity in the PI3K–PKB pathway and glucose uptake. Incubation of C_2_C_12_ myotubes with sAPPα increased phosphorylated PKB (Fig. 6d); this outcome was not replicated by sAPPβ (Fig. 6e). Furthermore, sAPPα increased glucose uptake (Fig. 6f), whereas sAPPβ had no effect (data not shown). The sAPPα-mediated glucose uptake was additive to that of insulin (Fig. 6g), as demonstrated for M-3 and APP overexpression. Taken together, these data suggest that modification of skeletal muscle APP cleavage by α- and β-secretases results in altered sAPPα abundance, which affects PI3K–PKB signalling to alter GLUT4 translocation and glucose uptake.

## Discussion

Inhibition of BACE1 in rodent myotubes increases glucose uptake and GLUT4 translocation independently of, but additive to, insulin-stimulated glucose uptake. Accordingly, reduced BACE1 activity increases insulin-stimulated glucose uptake in agreement with our previous study [21]. Impairment of insulin-stimulated glucose uptake by skeletal muscle is recognised as an early defect in the pathogenesis of type 2 diabetes, although the ability of alternative stimuli such as exercise/contraction to increase glucose uptake is unaffected [33]. However, non-insulin-dependent (basal) glucose uptake is also impaired in patients with type 2 diabetes. In the fasted state, when plasma insulin levels are low and skeletal muscle glucose uptake is insulin-independent, muscle glucose uptake is decreased in insulin-resistant individuals [34, 35]. Consequently, a novel pharmacological approach to increase glucose disposal by targeting basal skeletal muscle glucose uptake may have therapeutic utility.

Skeletal muscle glucose uptake occurs predominantly via the insulin-sensitive transporter, GLUT4, although GLUT1 and GLUT12 are also expressed with GLUT1 and thought to contribute to basal glucose uptake [36]. BACE1 inhibition did not alter glucose transporter mRNA or *HkII* mRNA or protein levels, although a small increase in GLUT1 and GLUT4 protein levels was observed. Nevertheless, the major effect of BACE1 inhibition was increased cell surface GLUT4myc in the absence of insulin. The increased basal and insulin-stimulated glucose uptake and GLUT4 translocation elicited by BACE1 inhibition was prevented by wortmannin, indicating a PI3K-regulated mechanism. Consequently, it is likely that the increase in basal glucose uptake observed following BACE1 inhibition is predominantly due to enhanced translocation of GLUT4, through activation of the canonical class 1A PI3K pathway [32].

Although we have not completely delineated the mechanism by which BACE1 modulates glucose uptake, our results indicate a key role for APP-cleavage products. APP membrane processing occurs predominantly by α-secretases (the ‘non-amyloidogenic’ pathway), most likely ADAM10 [37]. This ectodomain-shedding process liberates a soluble truncated form of APP, sAPPα. In contrast, BACE1 (the ‘amyloidogenic’ pathway) cleaves APP at a different site and releases a shorter soluble APP isoform, sAPPβ. Thus α- and β-secretases compete for APP cleavage, with the α-secretase pathway prevailing. Nevertheless, events such as chronic stress raise BACE1 activity and increase sAPPβ levels with a compensatory decline in sAPPα. Our results strongly indicate a role for the α-secretase pathway in modulating glucose uptake because: (1) α-secretase inhibition reduces glucose uptake in conjunction with diminished sAPPα in the medium; (2) APP overexpression (thus increased sAPPα) enhances basal and insulin-dependent glucose uptake; and (3) direct application of sAPPα increases basal and insulin-dependent glucose uptake and PI3K signalling, whereas sAPPβ does not affect either process.

Therefore we suggest that constitutive α-secretase activity maintains basal glucose uptake in muscle cells, whereas increased BACE1 activity inhibits this process by diverting APP down the amyloidogenic pathway. This reduces sAPPα in the medium and diminishes PI3K-driven GLUT4 translocation. Indeed, sAPPα exhibits neuroprotective properties and increases PI3K–PKB signalling and glucose uptake in neurons, effects not replicated by sAPPβ [38, 39]. These actions have been attributed to the C-terminal part of sAPPα, which differs from sAPPβ by the presence of an additional 16 amino acids. Thus it is likely that this region is also responsible for the increased glucose uptake and PI3K signalling in skeletal muscle. The receptor by which sAPPα may mediate these effects is at present unclear. However, sAPPα has structural similarities to cysteine-rich growth factors [40] and its neuroprotective function has been linked with activation of IGF-1 receptor/insulin receptor PI3K–PKB signalling in neurons [38].

The increased fat supply associated with obesity is a primary drive for the induction of skeletal muscle insulin resistance. Fatty acids are an important fuel source for muscle, and excess long-chain fatty acids, particularly saturated ones, in skeletal muscle raise levels of the lipid intermediates, diacylglycerol and ceramide, which are strongly implicated in the pathogenesis of insulin resistance [41, 42]. The skeletal muscle accumulation of diacylglycerol and ceramide is associated with impaired insulin signalling predominantly via the PI3K–PKB pathway [42, 43]. Exposure of C_2_C_12_ myotubes to palmitate or ceramide depressed insulin-dependent and -independent glucose uptake in the presence of BACE1 inhibitor. Thus the molecular mechanism underlying BACE1 action, which requires PI3K activity, is also sensitive to ceramide. Indeed, skeletal muscle BACE1 activity is increased by high-fat diet [21], and the finding that ceramide, concomitant with high BACE1 activity, strongly suppresses basal and insulin-dependent glucose uptake fits with the idea that lowering of sAPPα reduces non-insulin-dependent glucose uptake in muscle. Thus the balance between α- and β-secretase activities may be an important checkpoint in the control of skeletal muscle metabolism. Accordingly, pharmacological reduction of BACE1, or increased α-secretase activity, may represent a novel and reasonable therapeutic target to improve glucose uptake in tissues exposed to excess lipid.

BACE1 inhibition is also associated with increased glucose oxidation in C_2_C_12_ myotubes, with an increased proportion (over palmitate) of glucose oxidised for the same amount of oxygen consumed. Such an action in vivo would raise postprandial glucose uptake and oxidation and increase overall glucose disposal. Conversely, overexpression of BACE1 inhibited glucose oxidation and oxygen consumption with no reduction in palmitate oxidation. Furthermore, inhibition of α-secretase with batimastat reduced the increase in OCR elicited by palmitate in the presence of glucose, indicating diminished metabolic flexibility. Impairment of resting and insulin-stimulated mitochondrial oxidative phosphorylation has been reported in skeletal muscle of patients with type 2 diabetes [44, 45], and reduced glucose oxidation, with unchanged lipid oxidation, has been found to be associated with loss of metabolic flexibility in obese individuals [46]. At present, the molecular mechanism whereby BACE1 reduces glucose oxidation is unknown, with sAPPβ an unlikely mediator from our results. A plausible candidate is one or more of the β-amyloid peptides resulting from the sequential cleavage of APP by BACE1 and γ-secretase, as these aggravate the diabetic phenotype of rodents [47, 48].

Taken together, our results indicate that preventing skeletal muscle BACE1 activity from increasing excessively in times of chronic nutrient stress may have important implications, and indeed offer new therapeutic avenues, for the maintenance of glucose uptake and oxidation and the preservation of metabolic flexibility.

## Abbreviations

AD: Alzheimer’s disease
APP: Amyloid precursor protein
BACE1: Beta-site APP-cleaving enzyme 1
EV: Empty vector
HKII: Hexokinase II
mBACE1: Mutant BACE1
MEM: Minimal essential media
OCR: Oxygen consumption rate
PI3K: Phosphoinositide 3-kinase
PKB: Protein kinase B
sAPP: Soluble APP
